# Molecular Evidence of the Inhibitory Potential of Melatonin against NaAsO_2_-Induced Aging in Male Rats

**DOI:** 10.3390/molecules26216603

**Published:** 2021-10-31

**Authors:** Maryam Baeeri, Tina Didari, Madiha Khalid, Solmaz Mohammadi-Nejad, Seyed Mojtaba Daghighi, Ramtin Farhadi, Mahban Rahimifard, Zahra Bayrami, Hamed Haghi-Aminjan, Roham Foroumadi, Mahdi Gholami, Mohammad Abdollahi

**Affiliations:** 1 Toxicology and Diseases Group, Pharmaceutical Sciences Research Center (PSRC), The Institute of Pharmaceutical Sciences (TIPS), Tehran University of Medical Sciences, Tehran 1417613151, Iran; didari.tina@gmail.com (T.D.); madihakhalid777@gmail.com (M.K.); sonejad@gmail.com (S.M.-N.); daghighimojtaba@gmail.com (S.M.D.); ramtinfarhadi@gmail.com (R.F.); mahban.rahimifard@gmail.com (M.R.); bayrami101@gmail.com (Z.B.); m_gholami2068@yahoo.com (M.G.); 2 Pharmaceutical Sciences Research Center, Ardabil University of Medical Sciences, Ardabil 5618953141, Iran; hamedhaghi.a@gmail.com; 3 Department of Pharmacology, School of Medicine, Tehran University of Medical Sciences, Tehran 13145784, Iran; rohamforoumadi@yahoo.com; 4 Department of Toxicology and Pharmacology, Faculty of Pharmacy, Tehran University of Medical Sciences, Tehran 1417614411, Iran

**Keywords:** aging, Klotho, Melatonin, sodium arsenite, TERT, TRADD

## Abstract

Arsenic (As) poisoning is widespread due to exposure to pollution. The toxic level of (As) causes oxidative stress-induced aging and tissue damage. Since melatonin (MLT) has anti-oxidant and anti-aging properties, we aimed to evaluate the protective effect of MLT against the toxicity of sodium arsenite (NaAsO_2_). Healthy male NMRI mice were divided into eight different groups. The control group received a standard regular diet. Other groups were treated with varying diets, including MLT alone, NaAsO_2_, and NaAsO_2_ plus MLT. After one month of treatment, biochemical and pathological tests were performed on blood, heart, and lung tissue samples. NaAsO_2_ increased the levels of TNF-α, 8-hydroxy-2-deoxy guanosine (8OHdG), malondialdehyde (MDA), reactive oxygen species (ROS), and high mobility group box 1 (HMGB1), increased the expression of TNF receptor type 1-associated death domain (TRADD) mRNA and telomerase reverse transcriptase, and decreased the expression of Klotho (KL) mRNA in both plasma and tissues. In contrast, MLT reduced MDA, ROS, HMGB1, lactate, and TNF-α enhanced the mRNA expression of KL, and suppressed the mRNA expression of the TERT and TRADD genes. Thus, MLT confers potent protection against NaAsO_2_- induced tissue injury and oxidative stress.

## 1. Introduction

Arsenic is a metalloid and the number one toxin on the United States Environmental Protection Agency (USEPA) list of prioritized contaminants [1]. It remains one of the most important pollutants in groundwater [2]. Its concentration in drinking water of more than 50 µg/L is not considered safe for public health, but such levels are still common in many countries [1]. It has also been detected in several cosmetics, skin, face, hair, and herbal products. It is absorbed directly into the bloodstream through the skin and then accumulates in the body, causing toxic effects in different organs [3]. The inactivation of approximately 200 enzymes causes arsenic’s acute and chronic toxicity; many of these are involved in cellular energy pathways and in DNA synthesis and repair. Nausea, vomiting, stomach pain, and diarrhea are examples of acute toxicity, while chronic toxicity can lead to multisystem diseases and cancer affecting multiple organs [4]. Arsenic is cytotoxic, mutagenic, and genotoxic because it causes oxidative DNA damage, reduces anti-oxidant enzymes, and generates reactive oxygen species (ROS) [5]. The activation of telomerase is a crucial step in the progression of human cancers. The telomerase reverse transcriptase (TERT) gene’s transcriptional repression of its catalytic portion keeps it silent in healthy cells [6,7]. As a clastogenic agent, arsenic reduces telomerase expression and telomere length, causing apoptosis, necrosis, and the generation of ROS, and induces cell death [8].

Melatonin (MLT), a hormone secreted by the pineal gland, plays an essential role in circadian regulation. It also has anti-oxidant, anti-aging, immunomodulation, and cancer-fighting effects [9,10]. MLT helps avoid cellular aging by scavenging radicals and reducing oxidative damage to mitochondria [10]. MLT also helps in the reduction of mitomycin-C-induced genotoxic damage in peripheral blood in rats [11]. MLT can inhibit breast cancer cell proliferation and angiogenesis, cancer cell invasion, and telomerase function [12]. MLT decreases telomerase activity in tumor cells and mRNA expression of the TERT and TR subunits, restoring telomerase function [13,14]. The evidence indicates that MLT exposure can play a role in preventing oxidative stress-induced aging. This study is designed to evaluate whether MLT treatment can protect against oxidative stress-induced aging triggered by sodium arsenite (NaAsO_2_). 

Arsenic causes genotoxicity in human peripheral blood, while co-treatment with MLT significantly reduces genotoxic measures [15] and brain tissue damage [16,17]. In vitro studies have shown that MLT decreases arsenite-induced autophagy and promotes mitochondrial biogenesis in primary cultured neurons [18]. MLT treatment reduces pro-inflammatory cytokines, suggesting that it can protect the CNS from arsenic-induced oxidative stress, DNA damage, and apoptosis [19]. MLT has also shown protective effects against arsenic-induced metabolic toxicity [20], arsenic-induced liver/kidney toxicity, and arsenic-induced testicular injury [21]. These studies reveal that MLT protects against arsenic-induced toxicity in blood, liver, kidney, and testicular tissues. This study aims to evaluate whether MLT treatment protects the heart and lung tissues against oxidative stress-induced aging triggered by sodium arsenite (NaAsO_2_) as a potential protective effect of MLT against arsenic-induced aging in these tissues has not yet been explored.

## 2. Results

### 2.1. Total Tissue Arsenic Using an Inductively Coupled Plasma Mass Spectrometer

The concentrations of As in the heart and lung tissue samples exposed to 1/2, 1/3, and 1/10 LD50 NaAsO_2_, with and without MLT treatment, are shown in Table 1. The total As tissue concentration was found to be increased in both heart and lung tissue in a dose-dependent manner compared to the control group. MLT treatment, on the other hand, was shown to lower As tissue concentration. 

### 2.2. Oxidative Stress Biomarkers 

Malondialdehyde (MDA) is known as one of the crucial markers for oxidative stress assessment. As a high-grade toxicant, this chemical compound is a byproduct of cellular lipid peroxidation [22]. Heart and lung samples were examined after treatment with different doses of NaAsO_2_ alone or combined with MLT to assess oxidative stress biomarkers. In both heart and lung samples, we found similar levels of oxidative stress with varying quantities of NaAsO_2_ (1/2, 1/3, and 1/10 LD50). There was no statistical difference between the MDA levels of the control and MLT groups. A significant dose-dependent increase in the MDA levels of the 1/10 (both *p* < 0.05), 1/3 (both *p* < 0.0001), and ½ LD50 (both *p* < 0.0001) NaAsO_2_ groups was observed, in contrast to the control and MLT groups. However, MLT treatment prevented the increase in NaAsO_2_-induced MDA levels in both heart and lung samples compared to the ½ LD50 NaAsO_2_ group. A significant decrease in MDA levels in the MLT + NaAsO_2_ group was observed. These findings indicate that exposure to MLT can help reverse higher oxidative stress levels caused by NaAsO_2_ (Table 2).

### 2.3. Reactive Oxygen Species 

Reactive oxygen species (ROS) belong to the oxygen derivates; they damage cellular functions and have toxic effects. ROS molecules are composed of one oxygen and several unpaired electrons during inflammation [23]. Heart and lung samples were examined after treatment with different doses of NaAsO_2_ alone or in combination with MLT to assess reactive oxygen species. In both heart and lung samples, we found similar levels of ROS with varying quantities of NaAsO_2_ (1/2, 1/3, and 1/10 LD50). There was no statistical difference between the ROS levels of the control and the MLT groups. A significant dose-dependent increase in the ROS levels of the 1/10, 1/3, and ½ LD50 NaAsO_2_ groups (all *p* < 0.0001) was observed, in contrast to the control and MLT groups. However, MLT treatment prevented the increase in NaAsO_2_-induced ROS levels in both heart and lung samples compared to the ½ LD50 NaAsO_2_ group. A significant decrease in ROS levels in the MLT + NaAsO_2_ group (all *p* < 0.0001) was observed. These findings indicate that exposure to MLT can help reverse the increase in reactive oxygen species levels caused by NaAsO_2_ (Table 2). 

### 2.4. Pro-Inflammatory TNF-α Cytokine

**Figure 1:**
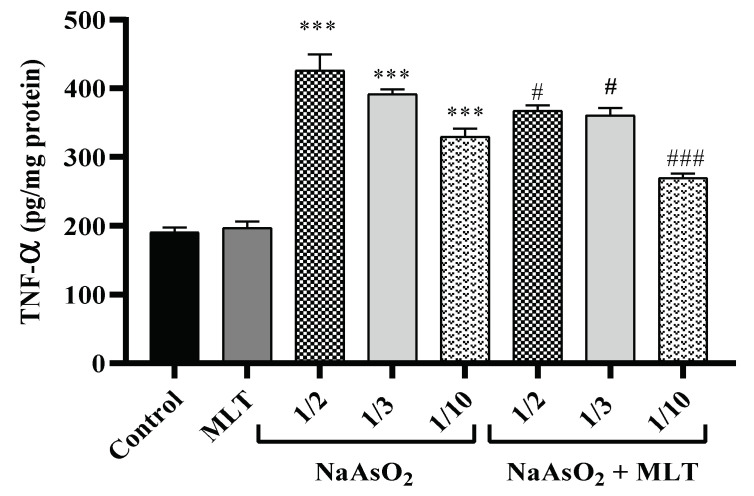
Assessment of pro-inflammatory TNF-α. Abbreviations: TNF-α: tumor necrosis factor-alpha; MLT; melatonin, NaAsO_2_; sodium arsenite; Results are expressed as mean ± SEM for five animals in each group (n = 5). *** *p* < 0.0001 (Vs. control or melatonin) ^###^ *p* < 0.0001; ^#^ *p* < 0.05 (Vs. ½ LD50 NaAsO_2_).

One of the essential pro-inflammatory cytokines in the early stage of inflammation is tumor necrosis factor-alpha (TNF-α). This cytokine is released from the macrophage when an inflammatory pathway is initiated, which causes cellular dysfunction. TNF-α is an immune system mediator in response to internal or external pathogens [24]. Plasma samples were examined after treatment with different doses of NaAsO_2_ alone or in combination with MLT for the assessment of TNF-α. There was no statistical difference in the TNF-α levels of the control and MLT groups. A significant dose-dependent increase in the TNF-α levels of the 1/10, 1/3, and ½ LD50 NaAsO_2_ groups (all *p* < 0.0001) was observed, in contrast to the control and MLT groups. However, MLT treatment prevented the increase in NaAsO_2_-induced TNF-α levels compared to the ½ LD50 NaAsO_2_ group. A significant decrease in TNF-α levels in the MLT + NaAsO_2_ group was observed. These findings indicate that exposure to MLT can help reverse the increase in TNF-α levels caused by NaAsO_2_ (Figure 1).

### 2.5. High Mobility Group Box 1 Levels

**Figure 2:**
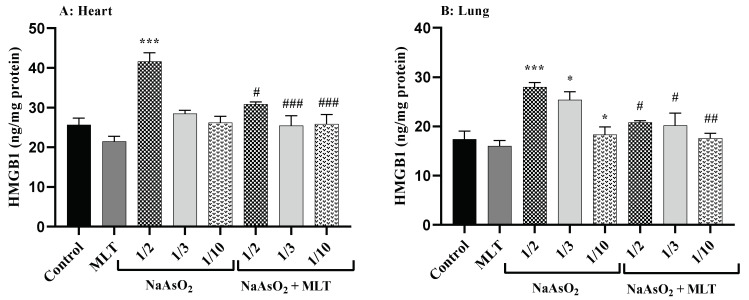
Assessment of High Mobility Group Box 1 (A) High Mobility Group Box 1 levels in heart; (B) High Mobility Group Box 1 levels in lung Abbreviations: HMGB1: High Mobility Group Box 1; MLT; melatonin, NaAsO2; sodium arsenite; Results are expressed as mean ± SEM for five animals in each group (n = 5). *** *p* < 0.0001; * *p* < 0.05 (Vs. control or melatonin). ### *p* < 0.0001; ## *p* < 0.001; # *p* < 0.05 (Vs. ½ LD50 NaAsO2).

High mobility group box 1 (HMGB1), a conserved nuclear protein and late mediator of inflammatory pathways, has a regulatory role in cellular damage during inflammation [25]. Heart and lung samples were examined for the level of HMGB1. No statistically significant difference was found between the HMGB1 levels of the control and the MLT groups. Compared to the control and MLT groups, a significant increase in HMGB1 levels in the ½ LD50 NaAsO_2_ group (both *p* < 0.0001) was observed. However, compared to the ½ LD50 NaAsO_2_ group, MLT treatment prevented the increase in NaAsO2-induced HMGB1 levels in both heart and lung samples. A significant decrease in levels of HMGB1 was observed in the MLT + NaAsO_2_ group. These results show that exposure to MLT will help reverse high levels of HMGB1 induced by NaAsO_2_ (Figure 2).

### 2.6. 8-Hydroxy-2-deoxyguanosine Levels

**Figure 3:**
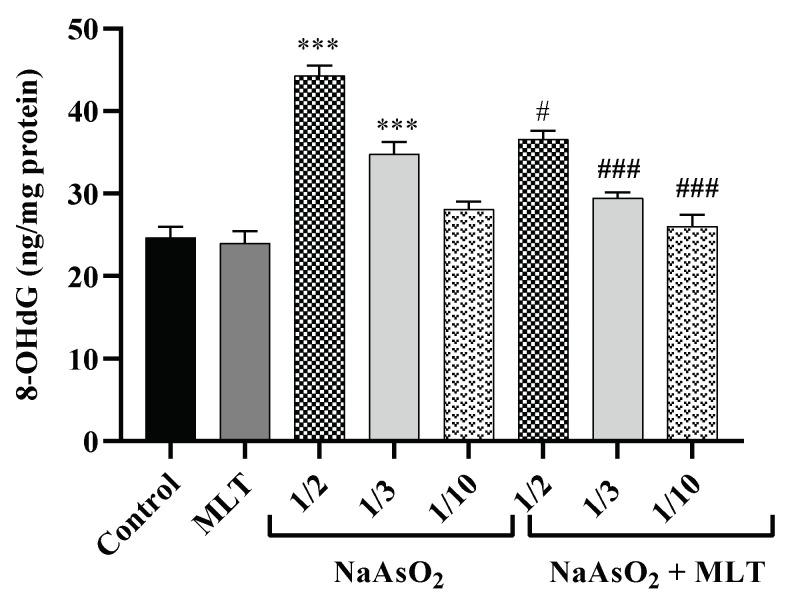
Assessment of 8-hydroxy-2-deoxyguanosine Abbreviations: 8OHdG: 8-hydroxy-2-deoxyguanosine; MLT; melatonin, NaAsO_2_; sodium arsenite; Results are expressed as mean ± SEM for five animals in each group (n = 5). *** *p* < 0.0001 (Vs. control or melatonin) ^###^ *p* < 0.0001; ^#^ *p* < 0.05 (Vs. ½ LD50 NaAsO_2_).

8-hydroxy-2-deoxy guanosine (8-OHdG) is a DNA oxidation product that establishes as an oxidative stress marker. Environmental factors affect the hydroxylation of guanosine [26]. Plasma samples were examined after treatment with different doses of NaAsO_2_ alone or combined with MLT to assess 8OHdG. There was no statistical difference between the 8OHdG levels of the control and the MLT groups. Significant increases in 8OHdG levels in the 1/2 and 1/3 LD50 NaAsO_2_ groups (both *p* < 0.0001) were observed, in contrast to the control or MLT group. However, MLT treatment prevented the increase in NaAsO_2_-induced 8OHdG levels compared to the ½ LD50 NaAsO_2_ group. A significant decrease in 8OHdG levels in the MLT + NaAsO_2_ group was observed. These findings indicate that exposure to MLT can help reverse oxidative DNA damage caused by NaAsO_2_ (Figure 3). 

### 2.7. Lactate Levels

Lactate synthesis increases during impaired cellular function and elevation of anaerobic reactions. Elevated lactate level is an indicator of mitochondrial damage and tissue hypoperfusion [27]. Heart and lung samples were examined after treatment with different doses of NaAsO_2_ alone or with MLT to assess lactate. We found different lactate levels in heart and lung samples. There was no statistical difference between the lactate levels of the control and the MLT groups.

In contrast to the control or MLT group, a significant increase in lactate levels in the 1/10, 1/3, and ½ LD50 NaAsO2 groups was observed. However, MLT treatment prevented the increase in NaAsO_2_-induced lactate level in both heart and lung samples compared to the 1/10 LD50 NaAsO_2_ and ½ LD50 NaAsO_2_ groups (all *p* < 0.0001). A significant decrease in lactate levels in the MLT + NaAsO_2_ group was observed. These findings indicate that exposure to MLT can help reverse increased lactate levels caused by NaAsO_2_ (Table 3). 

### 2.8. Real-Time Reverse Transcription PCR for Gene Expression

**Figure 4:**
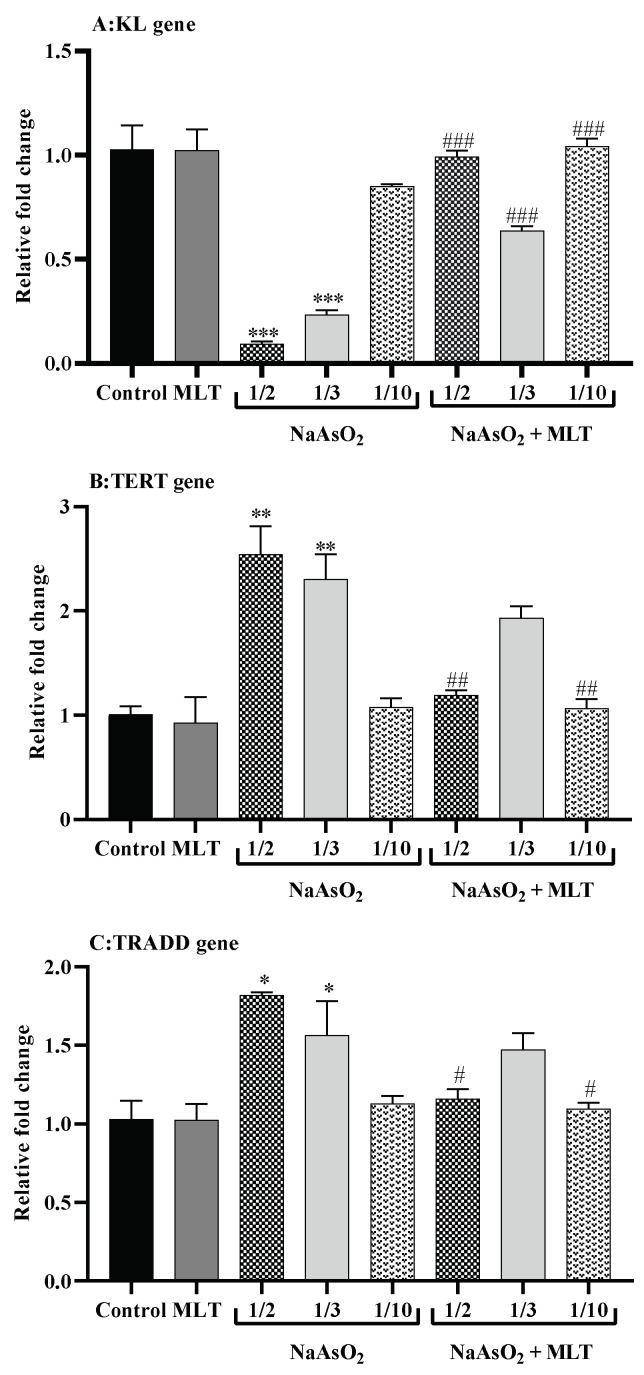
Quantitative real-time reverse transcription PCR for gene expression (**A**) Telomerase reverse transcriptase (TERT) gene expression; (**B**) Type 1 associated death domain tumor necrosis factor receptor (TRADD) gene expression; (**C**) Klotho related (KL) gene expression. Abbreviations: MLT; melatonin, NaAsO_2_; sodium arsenite; Results are expressed as mean ± SEM. Samples were analyzed in triplicate. *** *p* < 0.0001; ** *p* < 0.001; * *p* < 0.05 (Vs. control or melatonin) ^###^ *p* < 0.0001; ^##^ *p* < 0.001; ^#^ *p* < 0.05 (Vs. ½ LD50 NaAsO_2_).

In the presence of ½ (fold change 0.09) and 1/3 (fold change 0.23) LD50 of NaAsO_2_ (both *p* < 0.0001), the mRNA expression level of the anti-aging KL gene in the heart samples was significantly downregulated compared to the control (fold change 1.02) or MLT (fold change 1.02) groups (both *p* < 0.0001). On the other hand, MLT treatment tended to reduce the effects of NaAsO_2_, as demonstrated by the higher mRNA expression levels of KL genes in all treatment groups (all *p* < 0.0001) compared to the ½ LD50 NaAsO_2_ group. In the ½, 1/3, and 1/10 LD50 NaAsO_2_ + MLT treatment groups, the mRNA KL gene expression level increased compared to the ½ LD50 NaAsO_2_ group (All *p* < 0.0001). TERT is a telomerase catalytic subunit that, along with the telomerase RNA component (TERC), forms the fundamental unit of the telomerase complex. In normal cells, TERT gene expression is inactive or very low. In comparison to the control (fold change 1.00) and MLT (fold change 0.92) groups, we observed that exposure to ½ (fold change 2.54) and 1/3 (fold change 2.30) LD50 NaAsO_2_ (both *p* < 0.001) significantly doubled TERT gene mRNA expression. On the other hand, MLT exposure tended to minimize and normalize TERT overexpression in the treatment groups. The ½ and 1/10 LD50 NaAsO_2_ + MLT treatment groups had significantly decreased mRNA TERT gene expression (both *p* < 0.001). TRADD is a human adaptor protein responsible for programmed cell death and is encoded by the TRADD gene. Similar to TERT, in comparison to the control (1.03-fold) and MLT (1.02-fold) groups, we observed that exposure to ½ (1.81-fold) and 1/3 (1.56-fold) LD50 NaAsO_2_ (both *p* < 0.05) significantly increased TRADD gene mRNA expression. When compared to the ½ LD50 NaAsO_2_ group, MLT treatment reduced TRADD gene mRNA expression by 1.16 and 1.09-fold in the ½ and 1/10 LD50 NaAsO_2_ + MLT treatment groups (both *p* < 0.05) (Figure 4). 

### 2.9. Histological Evaluations of Heart and Lung Tissues 

**Figure 5:**
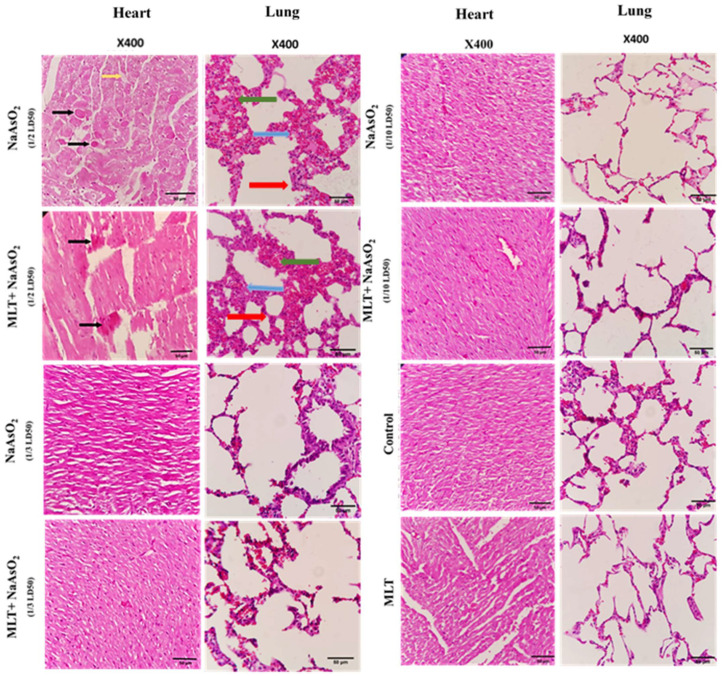
Hematoxylin–eosin-stained sections of tissues retrieved from animals treated with melatonin and arsenic in 1/2, 1/3, 1/10 doses, obtained at sacrifice after 30 days’ post-treatment. Black arrow refers to myocardial necrosis, Red arrow refers to inflammatory cells, Blue refers to Edema, Green arrow refers to hyperemia.

Representative results of the histological analysis of heart and lung tissues retrieved from rats treated with melatonin and NaAsO_2_ in 1/2, 1/3, 1/10 doses are presented in Figure 5. Any changes, including congestion, acute inflammatory response, necrosis, hemorrhage, or hyperemia, have been comparatively assessed in the different groups. Micrographs of heart and lung tissue from the 1/3 and 1/10 doses were normal without any histopathological changes. Histopathological evaluation of the group injected with the 1/2 dose of arsenic showed myocardial cell necrosis. However, in the 1/2 dose group, which also received melatonin, less necrosis of the heart tissue was apparent. When compared to the group that received melatonin and 1/2 arsenic in the lung, the group receiving only 1/2 arsenic showed more hyperemia, edema, and inflammatory cell infiltration.

## 3. Discussion

NaAsO_2_ is widely used in agriculture because of its antifungal, antibacterial, herbicidal, and rodenticidal properties. As a result of the widespread use of arsenic-based herbicides, pesticides, and livestock antibiotics [28]. On the other hand, MLT has shown anti-inflammatory, anti-oxidant, and anti-free radical effects [29]. Scavenging reactive oxygen and nitrogen species and boosting anti-oxidant defense prevents tissue damage and blocks transcriptional factors of pro-inflammatory cytokines [30]. MLT helps minimize arsenic-induced oxidative stress, as shown in this study. Exposure to the ½, 1/3, and 1/10 LD50 NaAsO_2_ caused an increase in the total As tissue concentration in both heart and lung tissues in a dose-dependent manner. MLT treatment, on the other hand, was found to lower As tissue concentration. MDA is a lipid peroxidation marker that plays a key role in the rise of oxidative stress by increasing ROS and early-stage inflammatory markers like TNF-α [31,32].

In contrast to the control and MLT groups, we found that NaAsO_2_ exposure caused a significant dose-dependent increase in MDA and, subsequently, ROS levels. MLT treatment was shown to reverse NaAsO_2_-induced MDA and ROS levels in both heart and lung samples. Our results are similar to those of Taysi et al., who identified that MLT could reverse oxidative injury caused by irradiation [33]. HMGB1 is an inflammatory cytokine that is released during the delayed phase of inflammation. We found the highest level of HMGB1 in both heart and lung tissues exposed to 1/2 LD50 NaAsO_2_, even after tissue extraction on the 30th day of analysis. MLT treatment, on the other hand, resulted in lower levels of HMGB1.

Similarly, plasma samples exposed to 1/2, 1/3, and 1/10 LD50 NaAsO_2_ showed elevated levels of TNF-α and 8OHdG, which was prevented by MLT, suggesting its role as a protector against oxidative DNA damage [34]. High lactate levels are used as a biomarker for tissue hypoperfusion and mitochondrial insufficiency [35]. After 30 days, there was an increase in lactate levels in both heart and lung tissues, indicating that NaAsO_2_ has a detrimental effect on mitochondria. On the other hand, MLT was found to defend against this effect, reducing lactate levels in both tissues. 

The protective effect of MLT was also confirmed by assessing KL, TERT, and TRADD mRNA expression levels. KL has been identified as a gene that plays a role in the aging process [36]. In rats, arsenite reduces circulating and renal KL and causes tubular damage [37]. In the heart tissue exposed to NaAsO_2_, we discovered a dose-dependent significant decrease (*p* < 0.0001) in the expression of mRNA KL, which was recovered after MLT treatment (*p* < 0.0001). Our results were similar to those of a study conducted on KL mutant mice, a genetic model of aging, in which MLT minimized oxidative stress and memory loss associated with KL deficiency [38]. The counteracting effect of MLT on aging was further confirmed in our study by the mRNA TERT gene expression level. In telomeres, which are strongly conserved and susceptible to age-dependent gradual attrition [39], NaAsO_2_ effectively doubled TERT gene mRNA expression in a dose-dependent manner. At the same time, MLT appeared to reduce and normalize TERT overexpression in the treatment groups, indicating that MLT counteracts NaAsO_2_-induced heart tissue aging. TRADD is a cell death gene that contributes to tissue aging by activating NF-кB and inducing apoptosis [40,41]. The apoptosis-inducing effect of NaAsO_2_ on heart tissues was confirmed by a significant increase in TRADD gene mRNA expression, which was decreased by MLT treatment. This indicates that MLT treatment restores regular TRADD gene mRNA expression and thus prevents heart tissue injury (Figure 5). 

In this study, the IP administration of both agents was preferred over the oral route to avoid the possible modification and degradation in the gastrointestinal tract. Additionally, IP administration provides faster and more reliable absorption than oral, ensuring optimal bioavailability of both small and large agents in rodent experimental studies [42]. 

Several studies and reviews indicate that melatonin produces significantly higher protective effects than the classic anti-oxidants [43]. In these studies, melatonin was found to be more effective than vitamin E [44,45,46], Beta-carotene [47], and Ascorbic acid [48,49]. Furthermore, in clinical settings, including chronic diseases such as rheumatoid arthritis [50], blood hypertension [51], and ischemia [52], beneficial anti-oxidant effects of melatonin in comparison to vitamins have been reported. Similarly, we found that exposure to NaAsO2 causes oxidative stress in the lungs and heart tissues dose-dependently in drug and chemical toxicity. At the high ½ LD50 NaAsO_2_ exposure level, TNF-α and 8OHdG in the plasma and MDA, ROS, and HMGB1 in the heart and lung tissues were elevated. These facts indicate that NaAsO_2_ exposure triggered oxidative stress in pulmonary and heart tissues by producing MDA and ROS and increasing the circulation of pro-inflammatory cytokines such as TNF-α. Tissue hypoperfusion, mitochondrial insufficiency, and inflammation may be caused by high tissue lactate and HMGB1 levels. Increased plasma 8OHdG levels suggest that NaAsO_2_ causes DNA damage.

According to the histological analysis, dose 1/2 of NaAsO_2_ in the heart tissues of rats caused cell necrosis correlated with the results for TERT and TRADD gene mRNA expression, showing a significant increase in the apoptosis-inducing effects of arsenic on heart tissues (Figure 4 and Figure 5). However, with the combined use of MLT and NaAsO_2_, necrosis clearance was much more potent and faster in MLT presence than in its absence. This clearance is evidenced by the decreased mRNA expression levels of the TERT and TRADD genes. Analogously, MLT attenuated the toxic effects of arsenic in histologically examined lung specimens, being correlated with the results obtained from inflammatory and oxidative stress biomarkers and the decrease in the score of hyperemia, edema, and inflammatory cells (Table 4). Furthermore, NaAsO_2_ exposure resulted in lower KL mRNA expression and higher TERT and TRADD mRNA expression, indicating a risk of heart tissue aging and cell death.

On the other hand, MLT treatment reduced MDA, ROS, HMGB1, and lactate levels in lung and heart tissues, as well as TNF-α and 8OHdG levels in plasma. It also boosted the mRNA expression of the KL gene while suppressing the mRNA expression of the TERT and TRADD genes. This indicates that MLT protects lung and heart tissues by reversing the risk of oxidative stress-induced aging triggered by NaAsO_2_. However, more research is needed to explore the protective role of MLT against arsenic-induced aging in other tissues such as the liver and kidney and the efficacy of MLT in lowering tissue arsenic levels.

## 4. Materials and Methods

### 4.1. Chemicals

MLT (CAS number 73-31-4), NaAsO_2_ (CAS number 7784-46-5), Suprapur HNO_3_ (Merck, Darmstadt, Germany), and all other chemicals used in this study were purchased from Sigma-Aldrich (GmbH, Munich, Germany). The Lactate isolation kit and ELISA kit for high mobility group box 1 (HMGB1) analysis were purchased from ZellBio GmbH (Ulm, Munich, Germany), and the TNF-α analysis kit was obtained from Diaclone (Besancon Cedex, France). Thermo Scientific Revert Aid first strand cDNA Synthesis Kit was obtained from Thermo Fisher (Vilnius, Lithuania). 

### 4.2. Ethical Approval 

Animal handling, care, killing, and other procedures involving animals were conducted in compliance with the Animal Research: Reporting of In Vivo Experiments (ARRIVE) guidelines. Biochemical and laboratory research and experiments were performed as per Good Laboratory Practices. The Research Committee at the National Institute for Medical Research Development (NIMAD) provided ethical approval for this study involving the use and treatment of animals under a code number: IR.NIMAD.REC.1397.542. 

### 4.3. Study Design 

**Figure 6:**
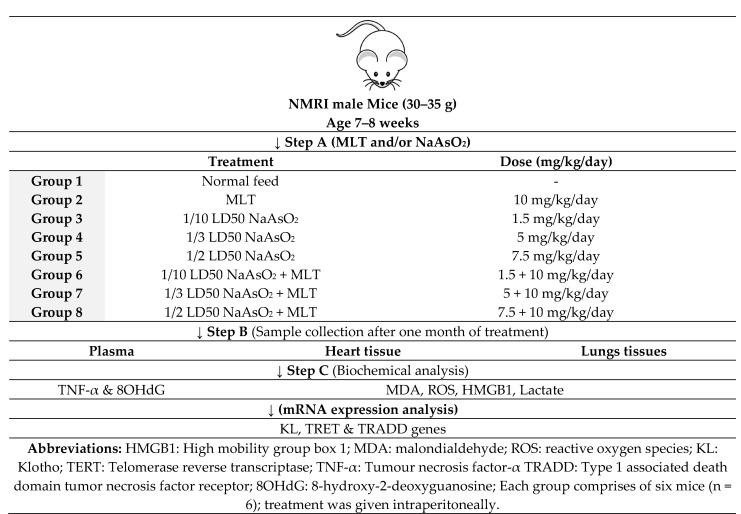
Study design.

In this study, we used male mice, not females, since the cyclic variations in the level of female hormones can influence toxicological studies. Thus, healthy and disease-free adult 30–35 g male NMRI mice aged 7 to 8 weeks were obtained from the animal house of the Faculty of Pharmacy of Tehran University of Medical Sciences, Tehran, Iran. They were kept under controlled environmental conditions, including room temperature of 20 to 25 °C, 50 to 55% relative humidity, and 12-h light and dark period. They had free access to a standard diet and water. The mice were divided into eight groups of six mice each. Group 1 received a standard normal diet. Group 2 received intraperitoneal (IP) injection of 10 mg/kg/day MLT, Group 3, 4 and 5 received 1.5 (1/10 LD50), 5 (1/3 LD50) and 7.5 (1/2 LD50) mg/kg NaAsO_2_, respectively. Groups 6, 7, and 8 were injected intraperitoneally with 1.5 (1/10 LD50), 5 (1/3 LD50), and 7.5 (1/2 LD50) mg/kg of NaAsO_2_ along with 10 mg/kg/day MLT, respectively during the last ten days of the experiment. After one month of treatment, IP injections of 100 mg/kg of ketamine and 10 mg/kg xylazine were used to kill the mice. Blood samples were collected in EDTA and serum separator tubes. The hearts and lungs were immediately removed and frozen at −80 °C for biochemical analysis. The tissue was collected from the heart and lung specimens for pathological examination. After washing with sterile phosphate buffer (pH 7.4), they were kept in 10 mL 10% formalin (Figure 6). 

### 4.4. Assessment of Total Tissue Arsenic Using an Inductively Coupled Plasma Mass Spectrometer 

The total arsenic in sample tissues was determined using an Inductively Coupled Plasma Mass Spectrometer (ICP-MS) (Perkin Elmer ELAN 6100 DRC-e). The instrument parameters are mentioned in the supplementary Table S1. Two mL phosphate buffer was added to 0.20 g of each sample, and the tissues were thoroughly homogenized. The homogenized tissues were transferred to glass test tubes and dried on a sand bath at 120 °C (approximately overnight). After the samples had reached room temperature, each tube was filled with 0.5 mL concentrated HNO_3_ and heated for two days. Temperatures did not exceed 120 °C during the heating process. Then 0.5 mL concentrated HNO_3_ was added to each test tube until white ash remained. After the digestion was completed and the solutions had cooled, 1 mL 10% HNO_3_ was added to each glass tube and sonicated for 30 min at 50 °C. The contents of the glass tubes were transferred to a 5 mL volumetric flask and calibrated to the sign with 10% HNO_3_. 10% HNO_3_ was used to make standard solutions with concentrations of 1, 5, 10, and 15 µg/L of As, and Germanium standard solution (5 g/L) was used as an internal standard solution [53].

### 4.5. Assessment of Oxidative Stress Biomarkers 

Thiobarbituric acid reactive substance (TBARS) assay was used to measure lipid peroxidation (LPO) activity by calculating levels of malondialdehyde (MDA) in heart and lung tissues. Heart or lung tissue samples of 0.15 g each were homogenized in phosphate buffer saline (PBS), blended with 800 mL of trichloroacetic acid, and centrifuged for 40 min at 3000 g. Then, 150 mL of 1% *w*/*v* Thiobarbituric acid was added to the 600 mL supernatant and placed in boiling water for 15 minutes. Finally, 400 mL of n-butanol was added, and a spectrophotometer was used to report absorbance at 532 nm [54]. 

### 4.6. Assessment of Reactive Oxygen Species 

ROS levels in heart and lung samples were calculated according to the previously described protocol [55]; 100 mg of each fresh heart and lung tissue sample were homogenized with an extraction buffer solution containing HEPES (5 mM), KCl (20 mM), EDTA, (1 mM), and sucrose (0.25 M), pH = 7.4 into each vial phosphate buffer and centrifuged at 10000 g for 10 min at 4 °C after adding the DL-dithiothreitol (DTT, 50 µM). In the next step, the supernatants of the heart and lung tissue samples were mixed with 80 µL of assay buffer and 5 µL of DCFH-DA (5 µM) for 15 min in 37 °C; 2′,7′-dichlorofluorescein diacetate (DCF-DA) is a cell-penetrating fluorogenic agent that converts into 2′,7′-dichlorodihydrofluorescein (DCFH). DCFH was then esterase-catalyzed intracellularly and ROS-oxidized into 2′,7′-dichlorofluorescein (DCF). Finally, the DCF absorbance was measured every 5 min by a multi-mode microplate spectrophotometer reader (BioTek^®^, Synergy^TM^ HT, Winooski, VT, USA) for 60 min with excitation and emission spectra of 488 nm and 525 nm, respectively. For standardizing data obtained from the ROS assay, each group of diluted tissue samples was used for determining the protein level. In summary, 10 µL Bradford reagent was added to 100 µL of diluted samples, and after incubation at room temperature for 15 min, the absorbance of each sample was read at 595 nm.

### 4.7. Assessment of Pro-Inflammatory Cytokines

Pro-inflammatory cytokine levels were evaluated, i.e., TNF-α in plasma and HMGB1 in heart and lung samples. These are recognized as the primary mediator of sepsis in the early and late phases [56]. HMGB1 level was performed for the heart and lung tissues, which were kept at −80 °C. An amount of 100 mg of each animal’s isolated heart and lung tissues was isolated and homogenized in phosphate buffer saline (PBS). The TNF-α and HMGB1 levels were quantified according to the manufacturer’s protocol. Absorbance was estimated at 450 nm. The TNF-α and HMGB1 levels were recorded in pg/mg protein and ng/mg protein, respectively. 

### 4.8. Assessment of DNA Damage

Eight-hydroxy-2-deoxyguanosine (8OHdG) is a product of oxidative damage to DNA. It is an essential biomarker for DNA damage. Plasma levels of 8-hydroxy-2-deoxy guanosine (8OHdG) were determined according to the 8OHdG ELISA kit (ZellBio GmbH [Ulm, Germany]). 

### 4.9. Assessment of Lactate Level

Tissue sample lactate levels were measured using a colorimetric lactate assay kit according to the provided protocol (ZellBio GmbH [Ulm, Germany]). In brief, 10 mL of perchloric acid 8% was homogenized with 100 mg of heart and lung samples. The standard curve was applied and recorded as mmol/mg of tissue protein to measure lactate levels. 

### 4.10. Quantitative Real-Time Reverse Transcription PCR for Gene Expression 

To detect the function of MLT and NaAsO_2_ in gene expression at the level of mRNA, quantitative real-time reverse transcription PCR (qRT-PCR) was used. Three genes associated with different molecular pathways, TERT, type 1 associated death domain tumor necrosis factor receptor (TRADD), and KL (related to Klotho activity), were evaluated in this respect. Tissue RNA was extracted using the RNX-Plus solution (SinaClon, Iran) protocol from frozen heart samples. RNA purity was spectrophotometrically calculated by Thermo Scientific NanoDrop (Thermo Scientific, Boston, MA, USA). The RNase-Free DNase I Kit was used to remove genomic DNA, then one microgram of extracted RNA was reverse transcribed to cDNA. Target genes have been normalized with the Glyceraldehyde 3-phosphate dehydrogenase (GAPDH) gene as an internal control. The LightCycler^®^ 96 System (Roche, Indianapolis, IN, USA) was used for qRT-PCR analysis. The SYBR green master mix was used, and qRT-PCR was performed under conditions, i.e., one cycle at 94 °C for 10 min, 40 cycles at 95 °C for 15 s, and annealing temperature for 30 and then 72 °C for 25 s. The results were reported according to the Pfaffle Method [57]. The specific primers used in the present study are shown in Table 5.

### 4.11. Histological Ex Vivo Evaluations

Heart and lung tissue samples were harvested after sacrificing the animals at day 30, while tissue samples were stored in 10 mL of 0.9% NaCl solution. The specimens were fixed in 10% neutral buffered formalin (NBF, PH. 7.26) for 48 h, embedded in paraffin, and cut into 5 µm sections. The sections were stained with hematoxylin-eosin and evaluated by an independent examiner using light microscopy (Olympus BX51; Olympus, Tokyo, Japan). 

### 4.12. Statistical Analysis

The results were presented as mean ± standard error of the mean (SEM). Statistical significance was assessed using a one-way analysis of variance (ANOVA) with statistical significance at *p* < 0.05.

## 5. Conclusions

**Figure 7:**
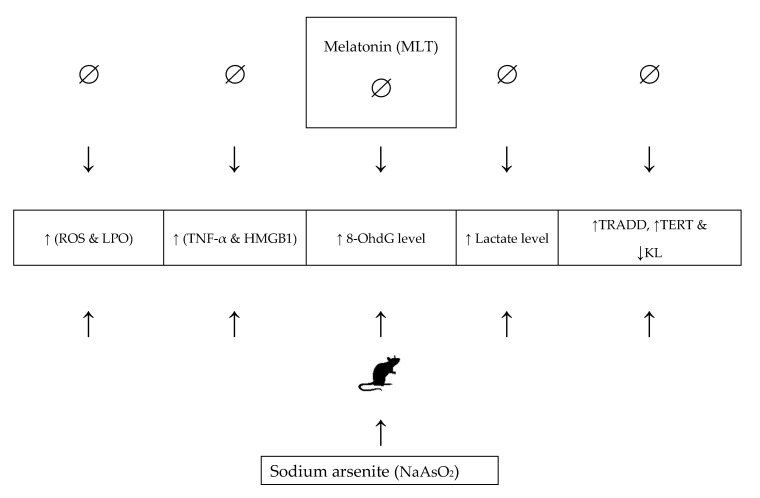
The role and mechanism of melatonin in alleviating NaAsO2-induced aging. Abbreviations: ROS: reactive oxygen species; LPO: lipid peroxidation; HMGB1: high mobility group box 1; TRADD: Tumour necrosis factor-α (TNF-α) receptor type 1-associated death domain; TERT: telomerase reverse transcriptase; KL: Klotho.

We conclude that MLT treatment can help reduce NaAsO_2_-induced oxidative stress-mediated aging. NaAsO_2_ causes oxidative stress, inflammation, DNA damage, and cell death in lung and heart tissues by raising MDA, ROS, lactate, HMGB1, TNF-α, and 8OHdG levels, which may lead to tissue aging and injury, as evidenced by decreased mRNA expression levels of KL induced by NaAsO_2_, and increased expression of the TERT and TRADD genes (Figure 7). MLT treatment, on the other hand, reduced the dose-dependent toxicity of NaAsO_2_. MLT has been shown to protect the lungs and heart from NaAsO_2_-induced tissue injury and aging by reducing the risk of oxidative stress, inflammation, DNA damage, and normalizing the mRNA expression levels of the KL, TERT, and TRADD genes.

## Figures and Tables

**Table 1:** Total As concentration in heart and lung tissues.

		Heart Tissue	Lung Tissue
Groups		Total As Concentration (mg/g)	Total As Concentration (mg/g)
Control		50 ± 3	25 ± 3
MLT		48 ± 2	23 ± 2
NaAsO_2_	1/2	475 ± 33 ***	425 ± 23 ***
	1/3	325 ± 25 ***	300 ± 23 ***
	1/10	200 ± 20 *	175 ± 18 *
NaAsO_2_ + MLT	1/2	375 ± 33	300 ± 25
	1/3	250 ± 15 ^#^	200 ± 8 ^#^
	1/10	175 ± 13 ^##^	100 ± 3 ^##^

Abbreviations: MLT; melatonin, NaAsO_2_; sodium arsenite. *** *p* < 0.0001; * *p* < 0.05 (Vs. control) ^##^ *p* < 0.001; ^#^ *p* < 0.05 (Vs. ½ LD50 NaAsO_2_).

**Table 2:** Assessment of oxidative stress biomarkers.

Oxidative Stress Biomarker					
		Heart Tissue		Lung Tissue	
Groups		MDA (µmol/mg protein)	ROS (mol/min/mg protein)	MDA (µmol/mg protein)	ROS (mol/min/mg protein)
	Control	0.71 ± 0.05	0.72 ± 0.05	0.50 ± 0.05	0.40 ± 0.05
	MLT	0.69 ± 0.05	0.69 ± 0.04	0.57 ± 0.04	0.42 ± 0.03
NaAsO_2_	1/2	1.67 ± 0.11 ***	1.87 ± 0.07 ***	1.46 ± 0.11 ***	1.50 ± 0.09 ***
	1/3	1.46 ± 0.06 ***	1.68 ± 0.06 ***	1.43 ± 0.09 ***	1.36 ± 0.06 ***
	1/10	1.14 ± 0.07 *	1.33 ± 0.03 ***	0.93 ± 0.07 *	1.01± 0.03 ***
NaAsO_2_ + MLT	1/2	1.30 ± 0.07 ^#^	1.05 ± 0.06 ^###^	1.09 ± 0.07 ^#^	0.65 ± 0.05 ^###^
	1/3	0.97 ± 0.09 ^###^	0.89 ± 0.05 ^###^	0.84 ± 0.06 ^###^	0.64 ± 0.08 ^###^
	1/10	0.88 ± 0.06 ^###^	0.74 ± 0.04 ^###^	0.62 ± 0.06 ^###^	0.43 ± 0.04 ^###^

Abbreviations: MDA: malondialdehyde; ROS: reactive oxygen species; MLT; melatonin, NaAsO_2_; sodium arsenite; Results are expressed as mean ± SEM for five animals in each group (n = 5). The ROS values are presented as (mol/min/mg protein). *** *p* < 0.0001; * *p* < 0.05 (Vs. control or melatonin) ^###^ *p* < 0.0001; ^#^ *p* < 0.05 (Vs. ½ LD50 NaAsO_2_).

**Table 3:** Assessment of lactate.

Lactate Level			
		Heart Tissue	Lung Tissue
Groups		Lactate (mmol/mg protein)	Lactate (mmol/mg protein)
	Control	0.73 ± 0.04	0.55 ± 0.04
	MLT	0.71 ± 0.09	0.51 ± 0.04
NaAsO_2_	1/2	1.30 ± 0.07 ***	1.34 ± 0.11 ***
	1/3	1.25 ± 0.04 ***	0.95 ± 0.04 *
	1/10	1.63 ± 0.06 ***	0.92 ± 0.05 *
NaAsO_2_ + MLT	1/2	0.96 ± 0.06 ^###^	0.83 ± 0.07 ^###^
	1/3	0.93 ± 0.04 ^###^	0.71 ± 0.03 ^###^
	1/10	1.07 ± 0.07 ^###^	0.65 ± 0.07 ^###^

Lactate levels in heart & lung tissuesAbbreviations: MLT; melatonin, NaAsO_2_; sodium arsenite; Results are expressed as mean ± SEM for five animals in each group (n = 5). *** *p* < 0.0001; * *p* < 0.05 (Vs. control or melatonin) ^###^ *p* < 0.0001 (Vs. 1/10 LD50 NaAsO_2_ in heart and Vs. ½ LD50 NaAsO_2_ in lungs).

**Table 4:** Histopathological scoring.

Histopathological Changes					
Groups		Edema	Congestion	Muscle Necrosis	Hemorrhage
Control		0.00 ± 0.00	0.00 ± 0.00	0.00 ± 0.00	0.00 ± 0.00
MLT		0.00 ± 0.00	0.00 ± 0.00	0.00 ± 0.00	0.00 ± 0.00
NaAsO_2_	1/2	3.33 ± 0.33 ***	2.33 ± 0.33 ***	4.67 ± 0.33 ***	2.00 ± 0.58 *
	1/3	0.33 ± 0.58 ^###^	0.00 ± 0.00 ^###^	0.67 ± 0.33 ^###^	0.67 ± 0.33
	1/10	0.00 ± 0.00 ^###^	0.00 ± 0.00 ^###^	0.00 ± 0.00 ^###^	0.00 ± 0.00 ^#^
NaAsO_2_ + MLT	1/2	2.33 ± 1.52 **	1.33 ± 0.33 ***^#^	3.00 ± 0.58 ***^#^	1.67 ± 0.67
	1/3	0 ± 0 ^###^	0.00 ± 0.00 ^###^	0.33 ± 0.33 ^###^	0.33 ± 0.33
	1/10	0 ± 0 ^###^	0.00 ± 0.00 ^###^	0.00 ± 0.00 ^###^	0.00 ± 0.00 ^#^

Abbreviations: MLT; melatonin, NaAsO_2_; sodium arsenite; Results are expressed as mean ± SEM for five animals in each group (n = 5). *** *p* < 0.0001; ** *p* < 0.001, * *p* < 0.05 (Vs. control or melatonin) ^###^ *p* < 0.0001; ^#^ *p* < 0.05 (Vs. ½ LD50 NaAsO_2_).

**Table 5:** The primers of genes used for RT-PCR.

Gene Name Primer	Gene Symbol	Accession No.	Primer Sequence (5′−3′)
**Mus musculus beta actin**	beta (Actb)	NM_007393.5	F: CAGCAAGCAGGAGTACGATGA R: TCAAAGAAAGGGTGTAAAACGCA
**Mus musculus TNFRSF1A-associated via death domain**	TRADD	NM_001033161.2	F: CGTGATGGGCTATACGAGCA R: CCGTGGGTTTCAAACACTGA
**Mus musculus klotho**	KL	NM_013823.2	F: GTTCTGCACTTCTACCGCTG R: GTGTTTGGCTCGTTCATGGT
**Mus musculus telomerase reverse transcriptase**	TERT	NM_001362388.1	F: CTGCAGGACACACCGTCTAT R: GTCACCTGTTGGTTTGCTGT

## Supplementary Materials

The following are available online. Table S1: Instrument parameters for inductively coupled plasma mass spectrometer (ICP-MS).

## Sample Availability

Samples of the compounds are not available from the authors.
