# Supplementary material for: Molecular Evidence of the Inhibitory Potential of Melatonin against NaAsO2-Induced Aging in Male Rats
Source: Molecules. 2021 Oct 31;26(21):6603. doi: 10.3390/molecules26216603 (PMC8587717; doi:10.3390/molecules26216603)
Supplement: Supplementary file 1 [file molecules-26-06603-s001.zip › molecules-1381535-supplementary.pdf]

molecules

MDPI

Supplementary Materials

Supplementary Table S1. Instrument parameters for Inductively Coupled Plasma Mass Spectrometer.

Parameter	Value	Unit
Nebulizer Gas Flow	0.69	L/min
ICP RF Power	1100	W
Lenz Voltage	6	V
Analog Stage Voltage	-2300	V
Pulse Stage Voltage	1600	V
